# Estrogen-Dominant Ovarian Cycle Stages Are Associated with Neural Network Dysfunction and Cognitive and Behavioral Deficits in the hAPP-J20 Mouse Model of Alzheimer’s Disease

**DOI:** 10.1523/ENEURO.0179-19.2019

**Published:** 2019-05-28

**Authors:** Rosalind S.E. Carney

## Abstract

**Highlighted Research Paper:** Ovarian Cycle Stages Modulate Alzheimer-Related Cognitive and Brain Network Alterations in Female Mice, by Lauren Broestl, Kurtresha Worden, Arturo J. Moreno, Emily J. Davis, Dan Wang, Bayardo Garay, Tanya Singh, Laure Verret, Jorge J. Palop, and Dena B. Dubal

Alzheimer’s disease (AD) is the most common form of dementia in older adults. In late-onset AD, initial symptoms such as memory impairment may first be noticed in individuals in their mid-sixties, whereas symptoms may be observed decades earlier in familial AD (National Institute on Aging, 2017). Seizures and altered neuronal network activity patterns, which may not cause overt cognitive defects, are thought to contribute to early AD pathogenesis in humans (Palop and Mucke, 2016; Vossel et al., 2017; Edwards and Robertson, 2018). Prospective cohort studies have shown that both familial and late-onset AD may be preceded by an asymptomatic preclinical phase that can last for decades. For example, reduced concentrations of amyloid-β (Aβ) in the cerebrospinal fluid and early onset of brain atrophy were found 15 years prior to the onset of AD symptoms (Bateman et al., 2012). Another longitudinal study found that Aβ deposition in the brain can occur over a protracted period of two decades or more (Villemagne et al., 2013). The untreated and significant pathophysiologic damage that occurs during the preclinical phase may explain why several Phase III clinical trials in older individuals with AD have been unsuccessful (Mehta et al., 2017). Without effective early intervention, it is estimated that up to 13 million people in the United States could be affected by AD by 2050 (Brookmeyer et al., 2011). Therefore, it is crucial to look at biological factors that could contribute to pathogenesis at the preclinical stage to delay disease onset and disease progression.

Broestl et al., 2018 hypothesized that the physiology of the female reproductive cycle could contribute to the preclinical pathogenesis of AD based on the following observations: (1) approximately two-thirds of individuals with AD are female; (2) the preclinical phase of AD likely overlaps with the reproductive life stage of women; (3) neural network dysfunction, such as hyperexcitability, and cognitive defects observed in AD have been linked to Aβ deposition in the brain (Palop and Mucke, 2010); and (4) the two primary ovarian hormones, estrogen and progesterone, can affect neuronal network activity. Estrogen can enhance neural function in non-disease states but increases susceptibly to seizures in mouse models of neurological conditions (Terasawa and Timiras, 1968; Woolley, 2000; Maguire et al, 2005; Ledoux et al. 2009). Conversely, susceptibility to seizures is reduced when progesterone levels predominate (Maguire et al, 2005; Wu et al., 2013). In their *eNeuro* publication, Broestl and colleagues examined whether the ovarian cycle of the transgenic hAPP-J20 (hAPP) mouse model of AD differs from that of non-transgenic (NTG) mice, and if so, could ovarian cycle alterations contribute to some of the early cognitive defects associated with AD pathogenesis.

The hAPP transgenic mouse expresses a variant form of the human amyloid precursor protein (hAPP) resulting from both the Swedish (K670N/M671L) and Indiana (V717F) mutations associated with familial AD (Mucke et al., 2000). Only female mice, matched by age and genetic background for each experiment and subjected to similar handling protocols, were used in the study; they had not yet developed amyloid plaques. First, the authors confirmed using vaginal cytology that hAPP mice exhibit all four stages of the mouse estrus cycle: proestrus, estrus, metestrus, and diestrus. When ovarian cycling was assessed over a three-week period, hAPP and NTG mice had the same number of estrous cycles of similar duration (each 4–5 d; one estrous cycle was defined as proestrus followed by estrus). However, based on known variations in the estradiol/progesterone ratio (E/P) during the estrous cycle (Walmer et al., 1992), Broestl and colleagues found that, compared to NTG mice, hAPP mice spent more time in the high E/P proestrus and estrus stages versus the low E/P stages of metestrus and diestrus (Fig. 1).

**Figure 1.**
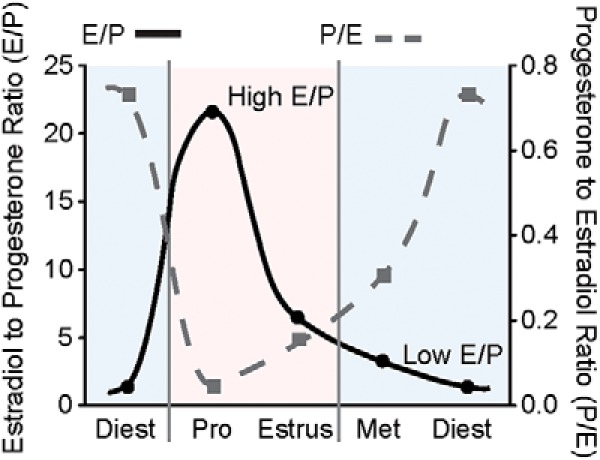
Altered ovarian cycling in female hAPP mice. Estradiol (pg/ml)-to-progesterone (ng/ml) ratio (E/P) and vice versa (P/E) at each stage of the mouse estrous cycle: diestrus (Diest), proestrus (Pro), estrus, and metestrus (Met). (Adapted from Fig. 1 in Broestl et al., 2018.)

Next, the authors tested whether high or low E/P stages could influence contextual fear memory task using a passive avoidance task. In an additional experimental group, the ovaries were removed (gonadectomized; Gnx) to examine the effects of ovarian hormone depletion in NTG (Gnx NTG) and hAPP (Gnx hAPP) mice. During testing, the latency to enter a dark chamber in which each mouse had received a foot shock 24 h prior was used as an indicator of contextual fear memory. The ability to learn the task was similar in all mice. However, the reduced latency during testing exhibited by hAPP mice in high E/P stages revealed significant memory defects compared to both hAPP mice in low E/P stages and NTG mice (Fig. 2). No significant statistical differences were found between the latencies of low E/P NTG and low E/P hAPP mice or Gnx NTG and Gnx hAPP mice. These results indicate that elevated levels of estradiol during the ovarian cycle adversely affect cognitive function in hAPP mice.

**Figure 2.**
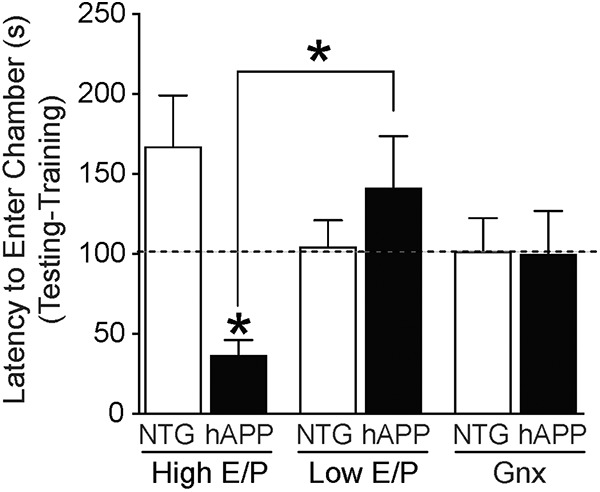
High E/P ovarian cycle stages, proestrus and estrus, worsen fear memory in female hAPP but not NTG mice. Latency (seconds) to enter the dark chamber during testing (minus latency during training) in NTG and hAPP female mice by ovarian status: high E/P (proestrus/estrus), low E/P (metestrus/diestrus), and Gnx NTG and Gnx hAPP mice. Data are mean ± SEM; * indicates *p* < 0.05 versus NTG or as shown by bracket. Dashed line indicates average of Gnx combined group. (Adapted from Fig. 3 in Broestl et al., 2018.)

Broestl and colleagues directly examined whether estrogen increases network hyperexcitability in Gnx mice. Gnx NTG and GNX hAPP mice were injected with a vehicle control or 17β-estradiol (E_2_) to replicate proestrus levels of estradiol. The latency to exhibit signs of seizure, induced 24 h later by treatment with GABA_A_ receptor antagonist pentylenetetrazole, was recorded later; a higher latency indicated increased resistance to seizure. E_2_ treatment led to a reduced latency to reach seizure and heightened seizure severity in Gnx hAPP mice compared to Gnx NTG mice. On the other hand, Gnx NTG and Gnx hAPP mice exhibited similar levels of seizure susceptibility in the vehicle control condition. These results further implicate the role of estrogen in the destabilization of neuronal networks and the hyperexcitability phenotype observed in hAPP mice.

The authors next determined whether Aß levels fluctuate during the ovarian cycle in hAPP mice. Aβ_1-42_ levels in the hippocampus of hAPP mice rose significantly during proestrus compared to other ovarian cycle stages (Fig. 3). There was no variation between *hAPP* mRNA or hAPP protein levels indicating that Aβ_1-42_ levels were affected post-translationally. Previous studies have shown that hyperexcitability affects APP processing and secretion of Aβ into the extracellular space (Kamenetz et al, 2003; Cirrito et al, 2005). Therefore, the estradiol-related increase in network hyperexcitability that occurs during the proestrus stage in hAPP mice could accelerate Aβ production in hAPP mice.

**Figure 3.**
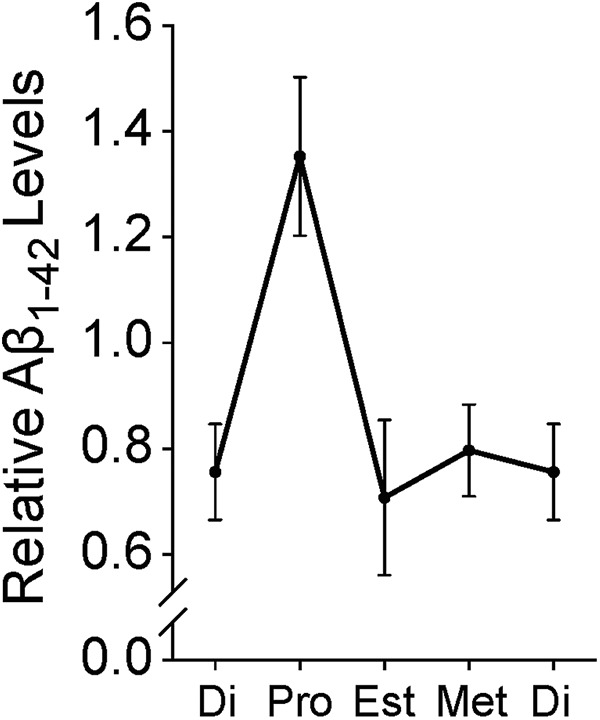
Levels of Aβ_1-42_ surge in the hippocampus of hAPP female mice during proestrus (Pro) compared to the other stages of the ovarian cycle, diestrus (Di), estrus (Est), and metestrus (Met). (Adapted from Fig. 6 in Broestl et al., 2018.)

Overall, these results show that hAPP mice exhibit previously unknown alterations in ovarian hormone levels that affect cognitive function. High E/P cycle stages in hAPP mice were associated with memory impairment, increased susceptibility to seizure, and elevated hippocampal levels of Aβ_1-42_. It would be interesting to determine whether women of reproductive age who may develop AD later in life have longer estrogen-dominant ovarian cycle stages, higher plasma Aβ levels, and cognitive impairments that may correlate with a surge in estradiol levels. Such observations could lead to potential early and even personalized interventions during the preclinical stage of AD. In addition, estrogen replacement therapy in post-menopausal women may accelerate pathophysiologic damage in women at risk for developing AD. This study also highlights the importance of reducing sex bias or sex omission in neuroscience research (Will et al., 2017). A recent study showed that firing patterns of hypothalamic gonadotropin-releasing hormone neurons vary according to ovarian cycle status in control mice and a mouse model of temporal lobe epilepsy (Li et al., 2018). Therefore, it may be important to consider ovarian cycle stage during cognitive and behavioral assessment in neurological disease states.
